# Negative Association between Serum Vitamin D Levels and Depression in a Young Adult US Population: A Cross-Sectional Study of NHANES 2007–2018 †

**DOI:** 10.3390/nu15132947

**Published:** 2023-06-29

**Authors:** Jiwen Ma, Ka Li

**Affiliations:** West China Hospital, West China School of Nursing, Sichuan University, 37 Guo Xue Rd., Chengdu 610041, China; majiwen@stu.scu.edu.cn

**Keywords:** American adults, depression, NHANES, serum vitamin D, cross-sectional study

## Abstract

Background: Vitamin D has been suggested to play a role in the development of depression, but the nature of the relationship between the two is still not fully understood. Although some studies have shown an association between vitamin D deficiency and depression, others have yielded inconsistent or inconclusive results. As a result, further research is needed to better understand the relationship between vitamin D and depression. Objectives: This study aims to assess the association between serum vitamin D and the risk of depressive symptoms in individuals aged 20 years and older in the United States. Methods: We selected 7562 participants from the 2007–2018 US National Health and Nutrition Examination Survey (NHANES). Participants’ serum vitamin D levels were determined from laboratory data, and those with a Patient Health Questionnaire-9 (PHQ-9) score of ≥ 10 were considered to have depressive symptoms. Associations between vitamin D and depressive symptoms were investigated using multiple logistic regression, subgroup analysis, and smoothed curve fitting. Results: In our study, 11.17% of the participants had depression. Multiple regression stratified analysis showed a significant inverse association between serum vitamin D and depression in the 29–39 years age group after full adjustment (OR = 0.54, 95% CI: 0.31–0.95, *p* = 0.0316). This result was supported by subgroup analysis and smoothed curve fitting. Conclusions: The association between serum vitamin D and depressive status in US adults varied across populations. Among those aged 29–39 years, vitamin D supplementation tended to show a lower rate of depression.

## 1. Introduction

Depression is the primary cause of the global burden of mental-health-related illness [1], and the third leading cause of disability in high-income countries, affecting around 840 million people worldwide [2]. One in six persons in the US experiences depression in their lives. Epidemiological studies indicate that one in four women and one in six men suffer from depression at some point in their lives, and it is more prevalent among younger individuals. Depression in older adults leads to increased morbidity and mortality, with a more pronounced effect in women than in men [3]. Depression is a mental health disorder that can range from mild to severe. The most common prodromal symptoms of depression include anxiety, nervousness, irritability, decreased energy, fatigue, sleep disturbances, and somatic discomfort [4]. By 2030, depression is expected to be one of the top three types of disease burden [5].

Vitamin D, also known as the “sunshine vitamin”, plays a crucial role in several physiological processes [6]. It is involved in various diseases such as osteoporosis, cancer, cardiovascular disease, diabetes, cognitive function, and mental health. Scientific literature has been extensively explored as proof to support vitamin D’s critical performance in many health conditions [7,8,9,10].

In recent years, the biological activity of vitamin D in humans and its role as a biomarker have garnered much attention. However, whether it can serve as a biomarker to reflect an individual’s level of mental health, such as depression status, remains unclear. Studies investigating the link between serum vitamin D concentrations and depression have reported inconsistent findings [7]. While some studies have established a link between vitamin D and depression [11,12,13,14], others have shown no link or have produced conflicting results. Furthermore, the results of existing meta-analyses on the relationship between serum vitamin D and depression are contradictory. Given this premise, there is a need to conduct cross-sectional studies with large sample sizes to explore the association between serum vitamin D and depression.

## 2. Materials and Methods

### 2.1. Data Sources and Study Population

Data for our study were obtained from the 2007–2018 National Health and Nutrition Examination Survey (NHANES) database, a nationally representative survey conducted by the National Center for Health Statistics (NCHS). The NHANES employs a complex multi-stage probability sample design to select a representative sample of noninstitutionalized civilians in the United States. The survey aims to evaluate the health and nutritional status of Americans and to facilitate epidemiological studies and health science research, which can contribute to the development of sound public health policy, guide the design of health programs and services, and advance the nation’s health knowledge [15,16,17,18].

**Figure 1:**
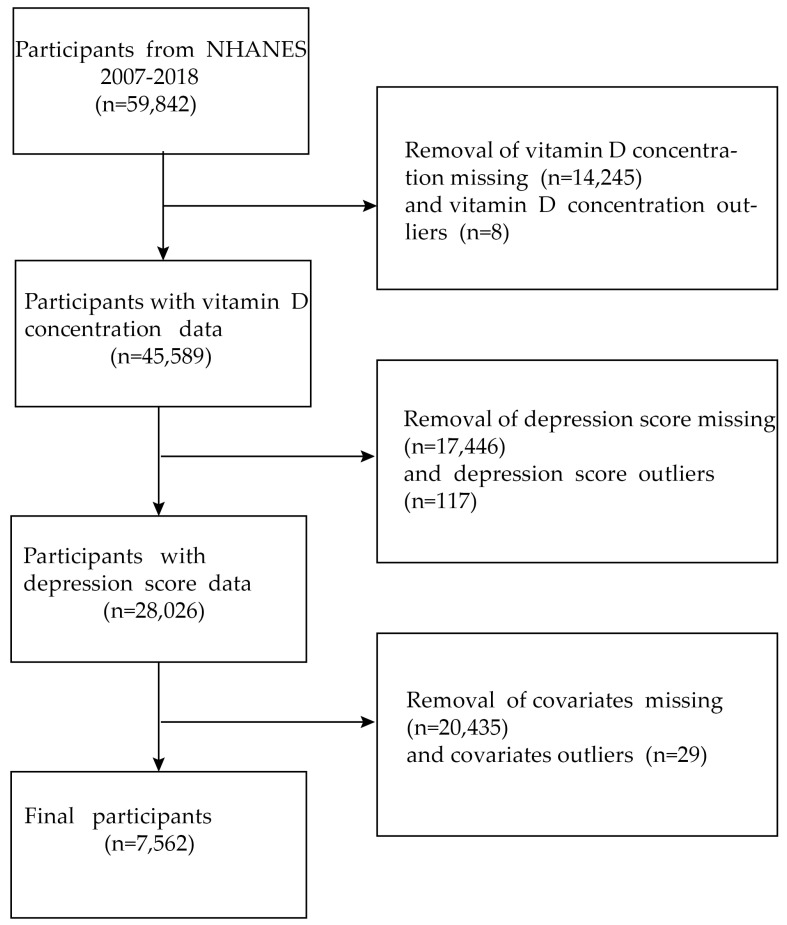
Flowchart for choosing participants. National Health and Nutrition Examination Survey, or NHANES.

Our study is a cross-sectional survey approved by the NCHS Ethics Review Board, and the original protocol is available online https://www.cdc.gov/nchs/nhanes/irba98.htm (accessed on 24 May 2023). All survey participants provided their informed consent before we gathered their demographic, nutritional, screening, laboratory, and questionnaire data. In total, 59,842 participants were enrolled in NHANES 2007–2018. After data cleaning, we excluded individuals with missing covariate data, vitamin D concentrations, depression questionnaire scores (PHQ-9), and outliers. Finally, our analysis dataset included 7562 US adults. Additional details regarding our study can be found on the official website (Available online: https://www.cdc.gov/nchs/nhanes/index.htm (accessed on 24 May 2023)). The procedure for picking samples is shown in Figure 1.

### 2.2. Determination of Serum Vitamin D Concentration

The determination of serum vitamin D concentration was achieved through the use of ultra-high-performance liquid chromatography-tandem mass spectrometry (UHPLC-MS/MS). This method allows for the quantitative detection of 25-hydroxyvitamin D3 (25OHD3), epi-25-hydroxyvitamin D3 (epi-25OHD3), and 25-hydroxyvitamin D2 (25OHD2) in human serum, which is considered the gold standard for assessing vitamin D status [19,20].

The serum samples were initially treated with an ethanolic solution containing three internal standards and a solution of 69–72% methanol, followed by the addition of hexane. The analytes were then extracted from the aqueous phase into the hexane layer via a liquid–liquid extraction process, which was then dried under a vacuum. The extract was re-dissolved using 69–72% methanol, and an aliquot of the extract was injected into the PFP column for the separation of 25OHD3, epi-25OHD3, 25OHD2, and the internal standards (IS), including 26,27-hexadeuterium-25-hydroxyvitamin D3, 6,19,19-tri deuterium-3-epi-25-hydroxyvitamin D3, and 6,19,19-tri deuterium-25-hydroxyvitamin D2. Thermo TSQ Vantage system’s MS-MS detects substances by using air pressure chemical ionization in the positive ion mode. Quantitation was achieved by comparing the peak area of the analyte in the unknown with the peak area of a known amount of analyte in a calibrator solution. Calculations were corrected based on the peak area of the internal standard in the unknown compared with the peak area of the matched internal standard in the calibrator solution. Please see the 2007 Experimental Methods at for further details on particular experimental techniques. (Available online: https://wwwn.cdc.gov/nchs/nhanes/continuousnhanes/labmethods.aspx?BeginYear=2007 (accessed on 24 May 2023)).

In our study, the term “serum vitamin D” refers to the concentration of 25OHD2+25OHD3 (nmol/L) in the blood. These represent the major metabolites of vitamin D, namely 25-hydroxyvitamin D2 and 25-hydroxyvitamin D3. Vitamin D exists in two main forms in the bloodstream: vitamin D2 and vitamin D3. When we consume vitamin D or expose our skin to sunlight, vitamin D2, and vitamin D3 are converted to 25-hydroxyvitamin D2 and 25-hydroxyvitamin D3 in the liver, and further metabolized into the active form, 1,25-dihydroxyvitamin D, in the kidneys. Measuring the levels of 25OHD2 and 25OHD3 in the serum provides an indication of an individual’s overall vitamin D status, encompassing both dietary intake and sunlight exposure, as well as the metabolic capacity of the liver and kidneys. Therefore, serum 25OHD2+25OHD3 levels are commonly used as an indicator of vitamin D status and metabolism.

### 2.3. Assessment of Depressive Symptoms

The assessment of depressive symptoms in this study was conducted using the Patient Health Questionnaire-9 (PHQ-9) program. Trained interviewers asked these questions at the Mobile Examination Center (MEC) using the Computer Assisted Personal Interview (CAPI) system, which has built-in consistency checks to minimize data entry errors. Additionally, the CAPI system includes online help screens to clarify any terms used in the questionnaire. To ensure the quality of the data, approximately 5% of the interviews were recorded and reviewed for quality control purposes. The PHQ-9 consisted of nine questions related to depressed mood, and the total score was calculated by summing the scores for each item. Response categories for the nine-item instrument, “not at all”, “several days”, “more than half the days”, and “nearly every day”, were given a point ranging from 0 to 3. The PHQ-9 scale had a range from 0 to 27, and scores were divided into the following categories: 0–4, normal, or no signs of depression. There are typically four categories of depression symptoms: mild (5–9), moderate (10–14), moderate to severe (15–19), and severe (20–27). A score of 10 or more was considered indicative of a depressive state [21,22,23,24]. The questionnaire can be found at https://wwwn.cdc.gov/Nchs/Nhanes/2013-2014/DPQ_H.htm (accessed on 24 May 2023).

### 2.4. Covariates

Based on the literature and clinical significance [7,22,24,25,26], this study utilized a variety of covariates, including gender, age, race, household income, education level, marital status, BMI, moderate recreational activity, sedentary activity in minutes, trouble sleeping, energy intake measured in kcal, waist circumference in centimeters, protein intake in grams, total sugars intake in grams, total polyunsaturated fatty acids intake in grams, vitamin E intake in milligrams, vitamin A intake in micrograms, vitamin B1 intake in micrograms, circumference in centimeters, vitamin B2 intake in milligrams, vitamin B6 intake in milligrams, total folate intake in micrograms, vitamin C intake in milligrams, alcohol consumption in grams, and smoking and drinking status. The study divided participants’ racial backgrounds into non-Hispanic White, non-Hispanic Black, other Hispanic, Mexican American, and other races. Education level was divided into two categories—below high school or above high school—and household income was calculated using the ratio of family income to poverty. There were two groups for marital status: with and without a partner. Each nutrient’s information was obtained from dietary data, while information on BMI (normal, overweight, and obese), along with waist circumference, was gleaned from examination data.

The study also utilized questionnaire data, which were self-reported by participants, to determine variables such as drinking habits, moderate recreational activity, sedentary time, and trouble sleeping.

### 2.5. Statistical Analysis

The STROBE criteria were strictly followed during the study’s execution. While categorical data appeared as frequencies and percentages, continuous variables were represented as means and standard deviations. R package 3.6.1 (http://www.R-project.org (accessed on 24 May 2023)) and EmpowerStats software (http://www.empowerstats.com (accessed on 24 May 2023)) were used to execute the statistical analyses. For continuous variables, the Kruskal–Wallis rank sum test was applied, and the Fisher exact probability test was applied for counting variables with theoretical numbers below 10. A 0.05 *p*-value was considered statistically significant.

We conducted a stratified analysis combining depression scores and serum vitamin D concentrations. The relationship between serum vitamin D levels and the likelihood of developing depressive symptoms was examined using multivariable logistic regression models. Model 1 was unadjusted, Model 2 was adjusted for age, gender, and race, and all variables were adjusted in Model 3, including gender, age, race, household income, education, marital status, BMI, waist circumference, moderate recreational activity, sedentary activity, trouble sleeping, energy intake, protein intake, total sugars intake, total polyunsaturated fatty acids intake, vitamin E intake, vitamin A intake, vitamin B1 intake, vitamin B6 intake, total folate intake, vitamin C intake, alcohol intake, drinking, and smoking status. To further explore the relationship between serum vitamin D and depressive symptoms, we performed smoothed curve fitting and subgroup analysis. A statistically significant difference was defined as a bilateral *p*-value of 0.05. The likelihood ratio test was utilized to look at interactions between subgroups.

## 3. Results

The total number of participants in this study was 7562, with a gender ratio of 60.10% and an average age of 49.22 ± 16.88 years. The median level of serum vitamin D was 65.58 ± 27.40 nmol/L. It was discovered that 11.17% of the individuals displayed depressive symptoms.

### 3.1. Baseline Characteristics of the Study Populations

Table 1 displays the characteristics of the study population stratified according to vitamin D groups in NHANES 2007–2018. The analysis reveals significant associations between vitamin D levels and various factors. Age and vitamin D levels show a significant correlation (*p* < 0.0001), as do household income and poverty ratio (*p* < 0.0001). Nutrient intake, including energy, protein, total sugar, and polyunsaturated fatty acids, differs significantly among vitamin D groups (*p* < 0.05). Similarly, vitamin E, vitamin A, vitamin B1, vitamin B2, vitamin B6, and total folate intake vary significantly based on vitamin D levels (*p* < 0.05). Demographic characteristics, such as gender (*p* < 0.0001) and race (*p* < 0.0001), also show significant differences across vitamin D groups. Educational attainment, marital status, body mass index, recreational activities, sedentary behavior, sleep problems, smoking, depression scores, and alcohol consumption exhibit significant variations (*p* < 0.05).

Table 2 displays the participants’ characteristics based on their depression scores in a study that included 7562 individuals, of which 845 scored 10 or more on the depression scale. The average age of participants with depression was 45.79 years, which was significantly lower than the average age of those without depression (49.65 years, *p* < 0.001). Additionally, the proportion of males in the non-depression group was significantly higher compared to that in the depression group (62.32% vs. 42.49%), while the proportion of females in the non-depression group was significantly lower than that in the depression group (37.68% vs. 57.51%, *p* < 0.001). Education, marital status, and most dietary variables also differed significantly between the two groups (*p* < 0.001).

### 3.2. Association between Serum Vitamin D Concentrations and Depressive Symptoms

Table 3 presents the results of the analysis investigating the association between vitamin D levels and depression scores. The unadjusted model shows a significant association between the two variables (OR = 0.99, 95% CI: 0.99–1.00, *p* < 0.0001). The adjustment I for gender, age, and race resulted in a significant association (OR = 0.99, 95% CI: 0.99–1.00, *p* = 0.0002). Adjustment II for additional factors, including income, education, marriage, BMI, lifestyle factors, and various nutrients, did not show a significant association (OR = 1.00, 95% CI: 1.00–1.00, *p* = 0.4140). However, stratified analysis revealed a significant association between vitamin D and depression in the age group of 29–39 years after adjusting for all covariates (OR = 0.54, 95% CI: 0.31–0.95, *p* = 0.0316). In adjusted model I, there was a more pronounced association observed between vitamin D levels and depression scores in women as opposed to men (OR = 0.57, 95% CI: 0.40–0.82, *p* = 0.0026). Furthermore, in adjusted model I, we observed a statistically significant association between the vitamin D concentration group of greater than 125 nmol/L and depression scores (OR = 0.45, 95% CI: 0.25–0.81, *p* = 0.0075). These results suggest that multiple factors may influence the association between vitamin D levels and depression scores.

Table 4 presents the results of a subgroup analysis that examines the association between vitamin D levels and depression scores, including subgroups such as gender, age, race, BMI, sedentary activity, moderate recreational activities, education, marital status, trouble sleeping, smoking, and drinking. The results show a significant interaction between vitamin D levels and age (*p*-value < 0.05). Specifically, among participants aged 29–39, the association between vitamin D levels and depression scores was stronger (OR: 0.99, 95% CI: 0.99, 1.00) compared to those aged 40–58 and 59–80 (OR: 1.00, 95% CI: 1.00, 1.01). Moreover, the results demonstrate a significant interaction between vitamin D levels and trouble sleeping (*p*-value < 0.05). Among participants without trouble sleeping, the association between vitamin D levels and depression scores was higher (OR: 0.99, 95% CI: 0.99, 1.00) compared to those with trouble sleeping (OR: 1.00, 95% CI: 0.99, 1.00).

**Figure 2:**
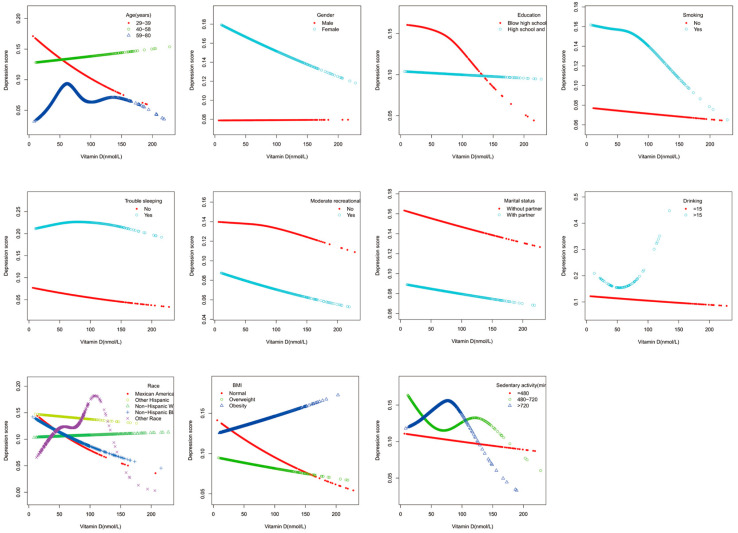
The associations between serum vitamin D (nmol/L) and depression scores were stratified by gender, age, race, BMI, education, marital status, sedentary time, moderate recreational activity, sleep disturbance, smoking, and drinking.

The results of smoothed curve fitting stratified by gender, age, race, BMI, education, marital status, sedentary time, moderate recreational activity, sleep disturbance, smoking, and drinking revealed that serum vitamin D was negatively associated with depression scores in the 29–39 age group and that serum vitamin D had more of an effect on depression scores in women than in men; see Figure 2.

## 4. Discussion

The present study utilized data from the NHANES 2007–2018 database, including complete serum vitamin D and Patient Health Questionnaire-9 (PHQ-9) data for 7562 U.S. adults. Serum vitamin D concentrations were quintile, and multiple regression was conducted to examine the association between serum vitamin D concentrations and the risk of depressive symptoms. Results indicated that serum vitamin D was negatively associated with the risk of depressive symptoms in U.S. adults aged 29–39 years, even after adjusting for potential confounders such as gender, age, race, the ratio of family income to poverty, education, marital status, BMI, waist circumference, moderate recreational activity, sedentary activity, trouble sleeping, energy, protein, total sugars, total polyunsaturated fatty acids, vitamin E, vitamin A, vitamin B1, vitamin B6, total folate, vitamin C, alcohol, drinking, and smoking. Smoothing curve fitting and subgroup analysis also supported this finding, demonstrating a tendency for improved mental state with increasing serum vitamin D concentrations in this age group. Furthermore, the study found that females were more likely to experience improvement in depressive symptoms with increased serum vitamin D levels compared to males.

Our research is compatible with prior studies [7,11,14,23,27]. Li et al. found a negative correlation between serum 25(OH)D3 levels and depressive symptoms, while Jorde et al. observed a relationship between serum 25(OH)D levels and depression, and suggested that high-dose vitamin D supplementation for one year may be beneficial for alleviating depressive symptoms. Furthermore, a meta-analysis of 65 randomized controlled trials and 31 observational studies demonstrated that vitamin D may play a significant role in the prevention and treatment of depression, particularly in individuals aged 50 or younger. Additionally, Ganji et al. discovered a higher prevalence of depression among individuals with vitamin D deficiency. Lastly, Hayley et al. reported notable gender differences in depression prevalence, with women experiencing depression at twice the rate of men.

In contrast to our findings, several studies have reported no significant benefit of vitamin D supplementation in improving depression [12,13,28,29], including the studies by Koning et al., Krivoy et al., Okereke et al., Zhu et al. Additionally, Rhee et al. found a negative association between low serum vitamin D levels and higher PHQ-9 scores in men, but not in women [22]. We suggest that the inconsistencies in results across studies may be due to differences in study settings and samples.

Vitamin D is a distinctive neurosteroid hormone, with its receptors present in various parts of the brain, including neurons and glia in the cingulate gyrus and hippocampus. Vitamin D plays a crucial role in numerous brain processes, such as the regulation of neurotrophic factors, neuroprotection, neuroplasticity, brain development, and neuroimmune regulation [2,29]. Insufficient vitamin D levels may be a potential contributing factor to depression [30].

Depression is believed to be caused by disruptions in the signaling between neurons, which may arise from various factors. One potential factor is microglia, which can lead to an imbalance in neuronal interactions, resulting in overstimulation of some neurons and over-suppression of others, ultimately leading to depressive symptoms [31]. Molecular pathway hypotheses have been proposed to explain the development of depression, including the monoamine neurotransmitter hypothesis, hypothalamic–pituitary–adrenal axis dysfunction hypothesis, neurotrophic factor hypothesis, and neuroinflammatory hypothesis [32]. Research has shown that the pathophysiology of depression is closely associated with changes in neurotransmitter levels, abnormalities in the hypothalamic–pituitary–adrenal axis, and/or inflammation [33].

The potential mechanisms underlying the relationship between vitamin D and depression are complex and multifaceted. One proposed indirect pathway is vitamin D’s role in promoting physical activity and well-being, which in turn may improve mood [11]. In addition, vitamin D may directly impact brain processes, such as synaptic plasticity and neural circuitry, which are involved in cognitive and emotional behavior [34]. Moreover, vitamin D is linked to microglia activation [35], and microglia have been implicated in the development of depression through the release of inflammatory factors and their direct effects on neurons [3]. Additionally, neuroinflammation may be a mediator between low serum vitamin D levels and depression, which could affect microglia and neurons in key brain regions such as the hippocampus, amygdala, and hypothalamus [6,36]. Finally, vitamin D may modulate intracellular calcium ion concentrations, which have been implicated in the pathogenesis of depression [31]. Overall, these complex and interrelated pathways suggest that vitamin D may play a multifaceted role in the etiology of depression.

Bioactive vitamin D primarily exerts its physiological functions by binding to the vitamin D receptor (VDR), which subsequently translocates to the nucleus and forms a heterodimer with the retinoid X receptor. This complex functions as a transcription factor by binding to the vitamin D response element (VDRE) in the target gene’s promoter region [37]. Research has suggested that VDR gene polymorphisms may impact the effectiveness of vitamin D in the brain, and individuals with certain variants may experience fewer depressive symptoms [25]. Vitamin D receptor expression is also increased in key regions of depression pathophysiology, and adequate vitamin D may have a neuroprotective function [38]. Furthermore, the activation of the STING-TBK1-IRF3 pathway has been shown to influence behavioral changes by enhancing microglia phagocytosis [39]. Conversely, vitamin D deficiency has been linked to reduced microglia phagocytosis and alterations in microglia morphology and function, potentially affecting neurosensory function [35,40]. Studies have also suggested that the STING-TBK1-IRF3 pathway is associated with depression, as its activation reduces neuroinflammation and promotes microglia phagocytosis, ultimately restoring the balance between these processes [39].

Our study has several strengths that contribute to the validity and reliability of our findings. First, we focused specifically on serum vitamin D levels and depressive symptoms, which is a relatively unexplored area in the literature. Additionally, our study used data from a representative national sample that was collected over five NHANES cycles, providing a broad and diverse dataset. We utilized the LC/MS/MS method, which is currently considered the gold standard for measuring serum vitamin D levels, and our measurements were subject to quality assurance and control by NCHS and NHANES staff. We also used a standardized instrument, the PHQ-9, to assess depressive status, which was shown to be reliable and valid in previous studies. Furthermore, we employed rigorous statistical methods, including multiple regression analysis and subgroup analysis, to adjust for potential confounding variables and to explore associations in different subgroups.

However, it is vital to recognize the limitations of our study. First, our assessment of depressive status relied solely on the PHQ-9 questionnaire and did not include a comprehensive psychiatric diagnosis. Second, we were unable to account for all potentially confounding variables, such as lifestyle factors, medication use, and other health conditions. Third, as with any cross-sectional study, we cannot infer causality from our findings. Last but not least, this complex functions as a transcription factor by binding to the vitamin D response element (VDRE) in the target gene’s promoter region.

In conclusion, our study provides important insights into the potential association between serum vitamin D levels and depressive symptoms, but further research is needed to confirm these findings and to determine the causal direction of this association.

## 5. Conclusions

Overall, our study provides evidence for an association between serum vitamin D levels and depressive symptoms in US adults, particularly in the age group of 29–39 years, with a negative correlation observed between the two variables. These findings suggest that maintaining adequate serum vitamin D levels may have a potential role in reducing the risk of depressive symptoms in this population. However, further large-scale prospective studies are needed to confirm these findings and to establish a causal relationship between serum vitamin D and depression.

## Figures and Tables

**Table 1:** Characteristics of the study population based on vitamin D groups in NHANES 2007–2018 (*n* = 7562).

Serum Vitamin D Groups	<30	≥30, <50	≥50, <125	≥125	*p* Value
Age (years)	43.21 ± 14.51	44.61 ± 15.60	47.86 ± 16.35	55.29 ± 14.50	<0.0001
Ratio of family income to poverty	2.11 ± 1.46	2.50 ± 1.63	3.07 ± 1.63	3.74 ± 1.52	<0.0001
Energy (kcal)	2288.62 ± 1188.03	2330.99 ± 1014.87	2309.18 ± 1013.64	2056.00 ± 864.00	0.0002
Protein (gm)	80.79 ± 48.61	85.01 ± 43.30	86.71 ± 42.63	81.01 ± 39.12	0.0070
Total sugars (gm)	111.66 ± 84.87	118.55 ± 82.18	115.88 ± 82.11	97.15 ± 66.11	0.0004
Total polyunsaturated fatty acids (gm)	18.23 ± 12.50	20.04 ± 12.70	20.23 ± 13.21	19.23 ± 12.43	0.0188
Vitamin E (mg)	7.22 ± 5.48	8.36 ± 5.98	9.10 ± 6.77	9.65 ± 6.20	<0.0001
Vitamin A (mcg)	472.08 ± 700.59	531.69 ± 486.83	653.67 ± 664.54	658.86 ± 421.46	<0.0001
Vitamin B1 (mg)	1.51 ± 0.97	1.60 ± 0.90	1.68 ± 0.93	1.49 ± 0.90	<0.0001
Vitamin B2 (mg)	1.84 ± 1.27	2.14 ± 1.41	2.38 ± 1.40	2.22 ± 1.31	<0.0001
Vitamin B6 (mg)	2.03 ± 1.59	2.18 ± 1.82	2.27 ± 1.69	2.16 ± 1.36	0.0236
Total folate (mcg)	369.93 ± 254.14	397.05 ± 254.41	417.56 ± 271.01	412.75 ± 253.15	0.0011
Vitamin C (mg)	67.45 ± 83.20	70.24 ± 87.64	73.61 ± 83.39	81.80 ± 79.89	0.0797
Waist circumference (cm)	103.21 ± 19.86	104.13 ± 18.41	99.95 ± 15.65	95.74 ± 14.40	<0.0001
Alcohol (gm)	27.48 ± 58.12	20.57 ± 38.12	19.86 ± 37.06	23.23 ± 32.36	0.0011
Gender					<0.0001
Male	52.14	60.99	57.72	29.45	
Female	47.86	39.01	42.28	70.55	
Race					<0.0001
Mexican American	9.78	13.83	5.27	1.06	
Other Hispanic	6.07	6.23	4.01	1.76	
Non-Hispanic White	33.13	51.86	81.17	89.83	
Non-Hispanic Black	41.90	20.33	4.20	3.78	
Other Race	9.11	7.75	5.35	3.58	
Education					<0.0001
Below high school	24.02	20.78	14.10	10.84	
High school and above	75.98	79.22	85.90	89.16	
Marital status					<0.0001
Without partner	53.36	42.82	35.62	30.98	
With partner	46.64	57.18	64.38	69.02	
BMI					<0.0001
Normal	27.58	23.69	29.48	35.72	
Overweight	25.89	27.44	35.26	38.29	
Obesity	46.53	48.87	35.26	25.99	
Moderate recreational activities					<0.0001
No	71.60	62.73	51.30	42.45	
Yes	28.40	37.27	48.70	57.55	
Sedentary activity (min)					<0.0001
≤480	58.82	58.40	65.66	63.09	
480–720	30.81	31.74	25.84	29.23	
>720	10.37	9.86	8.50	7.69	
Trouble sleeping					0.0263
No	69.68	69.01	66.09	61.46	
Yes	30.32	30.99	33.91	38.54	
Smoking					<0.0001
No	30.55	43.64	58.41	74.94	
Yes	69.45	56.36	41.59	25.06	
Depression score					0.0016
<10	87.12	87.72	90.46	93.07	
≥10	12.88	12.28	9.54	6.93	
Drinking					0.0327
≤15	97.93	98.78	99.11	99.93	
>15	2.07	1.22	0.89	0.07	

Mean ± SD for Age (years); Ratio of family income to poverty; Energy (kcal); Protein (g); Total sugars (g); Total polyunsaturated fatty acids (g); Vitamin E (mg); Vitamin A (mcg); Vitamin B1 (mg); Vitamin B2 (mg); Vitamin B6 (mg); Total folate (mcg); Vitamin C (mg); Waist circumference (cm); Alcohol (g). *p* value was calculated by the weighted linear regression model. % for: Gender; Race; Education; Marital status; BMI; Moderate recreational activities; Sedentary activity (min); Trouble sleeping; Smoking; Depression score; Drinking. *p* value was calculated by the weighted chi-square test.

**Table 2:** Characteristics of participants according to depression scores.

Depression Score	<10	≥10	*p*-Value
N	6717	845	
Age (years)	49.65 ± 17.04	45.79 ± 15.07	<0.001
Gender			<0.001
Male	4186 (62.32%)	359 (42.49%)	
Female	2531 (37.68%)	486 (57.51%)	
Race			0.107
Mexican American	842 (12.54%)	101 (11.95%)	
Other Hispanic	557 (8.29%)	92 (10.89%)	
Non-Hispanic White	3460 (51.51%)	411 (48.64%)	
Non-Hispanic Black	1283 (19.10%)	164 (19.41%)	
Other Race	575 (8.56%)	77 (9.11%)	
Education			<0.001
Below high school	1443 (21.48%)	251 (29.70%)	
High school and above	5274 (78.52%)	594 (70.30%)	
Marital status			<0.001
Without partner	2625 (39.08%)	475 (56.21%)	
With partner	4092 (60.92%)	370 (43.79%)	
The ratio of family income to poverty	2.56 ± 1.63	1.68 ± 1.38	<0.001
Energy (kcal)	2287.43 ± 1043.37	2169.70 ± 1050.51	<0.001
Protein (gm)	85.88 ± 44.10	75.90 ± 45.61	<0.001
Total sugars (gm)	115.09 ± 81.90	127.91 ± 94.98	<0.001
Total polyunsaturated fatty acids (gm)	19.86 ± 13.11	17.99 ± 13.12	<0.001
Vitamin E (mg)	8.64 ± 6.46	7.76 ± 6.61	<0.001
Vitamin A (mcg)	605.53 ± 629.37	509.24 ± 506.97	<0.001
Vitamin B1 (mg)	1.65 ± 0.93	1.48 ± 0.88	<0.001
Vitamin B2 (mg)	2.22 ± 1.37	2.08 ± 1.73	<0.001
Vitamin B6 (mg)	2.20 ± 1.62	1.99 ± 1.94	<0.001
Total folate (mcg)	408.48 ± 259.37	363.64 ± 245.67	<0.001
Serum Vitamin D (nmol/L)	66.07 ± 27.52	61.66 ± 26.10	<0.001
Vitamin C (mg)	76.69 ± 88.53	66.79 ± 91.94	<0.001
Alcohol (gm)	19.13 ± 36.98	16.03 ± 38.90	<0.001
Waist circumference (cm)	100.40 ± 16.02	102.67 ± 18.74	0.002
BMI			<0.001
Normal	1907 (28.39%)	233 (27.57%)	
Overweight	2338 (34.81%)	220 (26.04%)	
Obesity	2472 (36.80%)	392 (46.39%)	
Moderate recreational activities			<0.001
No	3848 (57.29%)	609 (72.07%)	
Yes	2869 (42.71%)	236 (27.93%)	
Sedentary activity (min)			0.004
≤480	4531 (67.46%)	524 (62.01%)	
480–720	1655 (24.64%)	235 (27.81%)	
>720	531 (7.91%)	86 (10.18%)	
Trouble sleeping			<0.001
No	4909 (73.08%)	325 (38.46%)	
Yes	1808 (26.92%)	520 (61.54%)	
Smoking			<0.001
No	3627 (54.00%)	286 (33.85%)	
Yes	3090 (46.00%)	559 (66.15%)	
Drinking			0.057
≤15	6647 (98.96%)	830 (98.22%)	
>15	70 (1.04%)	15 (1.78%)	

Mean ± SD/N (%). *p*-value: Kruskal–Wallis rank sum test for continuous variables, Fisher’s exact probability test for count variables with theoretical numbers <10.

**Table 3:** The associations between Vitamin D (nmol/L) and depression scores.

Exposure	Non-Adjusted	Adjust I	Adjust II
	OR (95% CI) *p*-Value	OR (95% CI) *p*-Value	OR (95% CI) *p*-Value
Serum Vitamin D (nmol/L)	0.99 (0.99, 1.00) <0.0001	0.99 (0.99, 1.00) 0.0002	1.00 (1.00, 1.00) 0.4140
Male	0.68 (0.46, 1.01) 0.0542	0.65 (0.42, 1.01) 0.0576	0.90 (0.56, 1.44) 0.6550
Female	0.46 (0.34, 0.64) <0.0001	0.57 (0.40, 0.82) 0.0026	0.95 (0.64, 1.42) 0.8022
Age (29–39)	0.52 (0.34, 0.81) 0.0041	0.44 (0.26, 0.74) 0.0018	0.54 (0.31, 0.95) 0.0316
Age (40–58)	0.79 (0.53, 1.18) 0.2550	0.68 (0.44, 1.06) 0.0863	1.16 (0.72, 1.89) 0.5422
Age (59–80)	0.71 (0.43, 1.18) 0.1911	0.75 (0.44, 1.28) 0.2846	1.13 (0.63, 2.03) 0.6842
Mexican American	0.39 (0.16, 0.94) 0.0351	0.41 (0.17, 0.99) 0.0477	0.46 (0.18, 1.19) 0.1094
Other Hispanic	0.78 (0.34, 1.80) 0.5565	0.63 (0.27, 1.49) 0.2942	0.78 (0.30, 2.06) 0.6220
Non-Hispanic White	0.51 (0.35, 0.75) 0.0007	0.56 (0.38, 0.84) 0.0043	1.23 (0.79, 1.92) 0.3524
Non-Hispanic Black	0.40 (0.21, 0.77) 0.0057	0.58 (0.29, 1.15) 0.1183	0.71 (0.33, 1.50) 0.3674
Other Race	1.28 (0.56, 2.93) 0.5605	1.28 (0.54, 3.05) 0.5797	1.56 (0.59, 4.12) 0.3674
Vitamin D (nmol/L) group			
<30	1.0	1.0	1.0
≥30, <50	0.87 (0.67, 1.14) 0.3176	0.95 (0.72, 1.25) 0.7106	1.04 (0.77, 1.40) 0.8006
≥50, <125	0.71 (0.56, 0.90) 0.0056	0.78 (0.60, 1.01) 0.0588	1.01 (0.76, 1.34) 0.9586
≥125	0.45 (0.25, 0.79) 0.0058	0.45 (0.25, 0.81) 0.0075	0.78 (0.42, 1.45) 0.4346

The non-adjusted model adjusts for: None. Adjust I model to adjust for Gender; Age (years); Race. Adjust II models adjust for Gender; Age (years); Race; Ratio of family income to poverty; Education; Marital status; BMI; Moderate recreational activities; Sedentary activity (min); Trouble sleeping; Energy (kcal); Waist. Circumference (cm); protein (gm); Total sugars (gm); Total polyunsaturated fatty acids (gm); Vitamin E (mg); Vitamin A (mcg); Vitamin B1 (mg); Vitamin B2 (mg); Vitamin B6 (mg); Total folate (mcg); Vitamin C (mg); Alcohol (gm); Drinking; Smoking.

**Table 4:** A subgroup of analyses for the association between Vitamin D (nmol/L) and depression scores.

Subgroup	N	OR (95% CI)	*p* Value	*p* (Interaction)
Gender				0.6639
Male	4545	1.00 (0.99, 1.00)	0.5797	
Female	3017	1.00 (0.99, 1.00)	0.1662	
Age (years)				0.0335 *
29–39	2507	0.99 (0.99, 1.00)	0.0213 *	
40–58	2521	1.00 (1.00, 1.01)	0.3275	
59–80	2534	1.00 (1.00, 1.01)	0.4810	
Race				0.3928
Mexican American	943	0.99 (0.98, 1.00)	0.1488	
Other Hispanic	649	1.00 (0.99, 1.01)	0.7461	
Non-Hispanic White	3871	1.00 (0.99, 1.00)	0.7181	
Non-Hispanic Black	1447	0.99 (0.99, 1.00)	0.1295	
Other Race	652	1.00 (0.99, 1.01)	0.3996	
BMI				0.2811
Normal	2140	1.00 (0.99, 1.00)	0.1487	
Overweight	2558	1.00 (0.99, 1.00)	0.5226	
Obesity	2864	1.00 (1.00, 1.01)	0.4757	
Sedentary activity (min)				0.8208
≤480	5055	1.00 (0.99, 1.00)	0.3741	
480–720	1890	1.00 (0.99, 1.00)	0.6005	
>720	617	1.00 (0.99, 1.01)	0.7524	
Moderate recreational activities				0.6966
No	4457	1.00 (1.00, 1.00)	0.6090	
Yes	3105	1.00 (0.99, 1.00)	0.4277	
Education				0.3304
Below high school	1694	1.00 (0.99, 1.00)	0.1643	
High school and above	5868	1.00 (1.00, 1.00)	0.6394	
Marital status				0.4464
Without partner	3100	1.00 (1.00, 1.00)	0.9027	
With partner	4462	1.00 (0.99, 1.00)	0.2687	
Trouble sleeping				0.0517
No	5234	0.99 (0.99, 1.00)	0.0012 **	
Yes	2328	1.00 (0.99, 1.00)	0.3067	
Smoking				0.6026
No	3913	1.00 (1.00, 1.01)	0.7082	
Yes	3649	1.00 (1.00, 1.00)	0.7144	
Drinking				0.4411
≤15	7477	1.00 (1.00, 1.00)	0.1919	
>15	85	1.01 (0.98, 1.04)	0.5222	

The results of subgroup analysis were adjusted for Energy (kcal); Ratio of family income to poverty; Protein (gm); Total sugars (gm); Total polyunsaturated fatty acids (gm); Vitamin E (mg); Vitamin A (mcg); Vitamin B1 (mg); Vitamin B2 (mg); Vitamin B6 (mg); Total folate (mcg); Vitamin C (mg); Alcohol (gm); Waist. Circumference (cm). 95% CI, 95% Confidence Interval; OR, Odds Ratio; BMI was categorized as normal (<25 kg/m^2^), overweight (25 kg/m^2^–29.9 kg/m^2^), and obese (≥30 kg/m^2^). *: *p*-value < 0.05; **: *p*-value < 0.01.

## Data Availability

Publicly available datasets were analyzed in this study. This data can be found here: NHANES database (https://www.cdc.gov/nchs/nhanes/index.htm (accessed on 24 May 2023)).
